# Loneliness, Social Isolation and Atrial Fibrillation: A Systematic Review From a Psychiatric Perspective

**DOI:** 10.1192/bjo.2026.11162

**Published:** 2026-06-30

**Authors:** Shriya Karlapudi

**Affiliations:** Faculty of Life Sciences and Medicine, King's College London, London, United Kingdom

## Abstract

**Aims:**

Social isolation and loneliness represent significant psychiatric determinants of cardiovascular disease, with emerging neurobiological evidence suggesting shared pathways for stress response and inflammation.

Social isolation reflects an objective lack of social contact while loneliness refers to the subjective perception of social disconnection. In practice, both frequently co-occur.

Current research inadequately examines the nuanced relationship between social disconnection and atrial fibrillation (AF) incidence. This paper aimed to systematically assess this relationship through a psychiatric lens.

**Methods:**

A PRISMA-compliant systematic review was conducted across five databases. Five studies were selected, including large population-based cohorts from the UK Biobank, the Framingham Heart study and a nationwide heart failure cohort. Data on social isolation, loneliness, social network metrics and AF incidence were extracted. Narrative synthesis was performed due to the heterogeneity of populations and analytical methods.

**Results:**

Large observational cohort studies suggest that loneliness is associated with an increased risk of AF. In the UK Biobank, loneliness assessed via a composite score was associated with higher AF incidence (HR: 1.11, 95% CI 1.07–1.16). Subscale analyses demonstrated that this association was primarily driven by subjective feelings of loneliness, which showed a stronger association with AF (HR 1.20, 95% CI 1.14–1.26). This is supported by multistate modelling in a separate Biobank study. Social deprivation was also correlated with new-onset AF in a nationwide heart failure cohort.

In contrast, the Framingham Heart Study reported no association between overall social network index and AF incidence. When accounting for the competing risk of mortality, medium-low social connectedness appeared to have a lower AF incidence. This finding is likely attributable to survivorship and competing risk biases rather than a true protective effect. Furthermore, social group participation was associated with higher AF incidence, which may reflect increased healthcare access and consequent AF detection.

Most importantly, Mendelian Randomisation analysis in one study provided genetic evidence supporting a potential causal relationship between social isolation and AF risk, with no evidence of pleiotropy.

Overall, these findings indicate that survivorship bias, varying analytical methods and failure to consider competing mortality risks may obscure true associations between social disconnection and AF incidence.

**Conclusion:**

Observational and genetic evidence support an association between social isolation and increased AF incidence. These findings highlight the need for novel AF prevention strategies such as psychosocial assessments and interventions to target loneliness and social disconnection, thereby emphasising the importance of a collaborative psychiatric-cardiology approach in clinical practice.

